# Clinical Characteristics and Outcomes of Patients With Primary Mediastinal Germ Cell Tumors: A Single-Center Experience

**DOI:** 10.3389/fonc.2020.01137

**Published:** 2020-07-16

**Authors:** Lu Wang, Jun Zhao, Tongtong An, Yuyan Wang, Minglei Zhuo, Meina Wu, Ziping Wang, Jianjie Li, Xue Yang, Hanxiao Chen, Jia Zhong

**Affiliations:** ^1^Department of Radiotherapy, Peking University Third Hospital, Beijing, China; ^2^Key Laboratory of Carcinogenesis and Translational Research (Ministry of Education), Department of Thoracic Medical Oncology-I, Peking University Cancer Hospital and Institute, Beijing, China

**Keywords:** primary mediastinal germ cell tumors, seminoma, nonseminoma, radiotherapy, prognosis

## Abstract

**Purpose:** Primary mediastinal germ cell tumors (PMGCTs) are rare. The natural history and optimal treatment strategies still need to be defined. The aim of the study was to summarize the clinical characteristics, treatment outcomes, and prognostic factors of PMGCTs.

**Methods:** Twenty-four patients with PMGCTs who were treated from December 2008 to January 2019 were evaluated retrospectively. The Kaplan–Meier method and Cox regression analysis were used to evaluate factors associated with prognosis.

**Results:** The study population consisted of 23 male patients and 1 female patient. Five patients were diagnosed with seminoma and 19 patients were diagnosed with nonseminoma. The median follow-up time for all patients was 15.8 (3.9–114.5) months. The 5-year overall survival (OS) and progression free survival (PFS) rates for all patients were 65.2 and 44.3%. For nonseminoma and seminoma, the 5-year OS rates were 54.1 and 100% (*P* = 0.093), respectively, and the 5-year PFS rates were 28.7 and 100%, respectively (*P* = 0.044). In patients with nonseminoma, first-line radiotherapy indicated superior OS and PFS (*P* = 0.037 and 0.027, respectively). The median survival time after recurrence was 4.3 months and the 1-year survival rate after recurrence was 23.4%.

**Conclusion:** These results indicated that in PMGCTs, the prognosis of seminoma is superior to that of nonseminoma. Radiotherapy may be an essential treatment in patients with nonseminoma. Patients with relapse have unfavorable prognosis.

## Introduction

More than 90% of germ cell tumors (GCTs) originate from the testis. And only 2–5% of GCTs are primary extragonadal germ cell malignancies, which are predominantly found in mediastinum and retroperitoneum (1). Their histologies are identical with their testicular counterparts and can be divided into seminoma and nonseminoma (2).

The genesis of extragonadal germ cell tumors (EGCTs) is still unknown. There are two prominent theories. One proposes that germ cell precursors migrate improperly and survive in ectopic locations. The second believes that the tumors develop from germ cells that are widely distributed during embryogenesis (3, 4).

PMGCTs are relatively rare and only account for 1–3% of all adult GCTs (5). Primary mediastinal seminoma develops relatively slowly and occultly. Primary mediastinal nonseminoma develops faster and at least one metastatic lesion is present in 85–95% of patients when they are diagnosed for the first time (4, 6). Furthermore, nonseminoma is more likely to metastasize than seminoma (7). More than 90% of patients have symptoms due to giant mass or invasion (8). These symptoms are usually nonspecific with cough, dyspnea, hoarseness, dysphagia, and chest pain being the most common. Besides, some patients may develop symptoms, such as the superior vena cava syndrome, weight loss, fever, and fatigue (4).

PMGCTs share the same serum tumor markers (STMs) with gonadal GCTs, such as lactate dehydrogenase (LDH), alpha-fetoprotein (AFP), and beta-human chorionic gonadotropin (β-HCG). Additionally, a characteristic genetic abnormality, named isochromosome 12p, can be found both in extragonadal GCTs and gonadal GCTs (9). The computed tomography (CT) image characteristics of PMGCTs are similar to that of gonadal GCTs. However, variation can be observed on CT between seminoma and nonseminoma. Primary mediastinal seminoma shows homogeneous soft tissue attenuation and homogeneous enhancement after contrast administration. Whereas, primary mediastinal nonseminoma is heterogeneous with irregular borders, which may extend into adjacent structures (10, 11).

To our knowledge, there are few PMGCT studies with large numbers of patients and no consensus about the standard treatment modality of PMGCTs. Additionally, the reports of Asian PMGCTs in English literature fewer still. Some studies reported that the survival rate of oriental PMGCT patients was inferior to that of occidental patients (12), an observation that warrants further investigation. Our aim of the study was to retrospectively analyze the efficacy of treatments and prognostic factors of Chinese patients with PMGCTs.

## Patients and Methods

### Data Collection

The data of 24 patients who were treated from December 2008 to January 2019 consecutively in Peking University Cancer Hospital were enrolled in the study. All the patients were diagnosed as PMGCT and patients with GCT originating from the gonad or other extragonadal sites were excluded from the study. Patients' baseline information (age, sex, location of the tumor, tumor size, histology, and serum tumor markers including β-HCG, AFP, and LDH), treatment strategies and efficacy were evaluated retrospectively. All data was obtained and recorded anonymously by our coinvestigators. This study was reviewed and approved by the Institutional Ethics Committee of Peking University Cancer Hospital (2019KT66).

Tumor response was accessed by oncologists according to RECIST 1.1 (13). Complete response (CR) was defined as disappearance of all target lesions. Partial response (PR) was defined as at least a 30% decrease in the diameters of the target lesions. Progressive disease (PD) was defined as at least a 20% increase in the diameters of the target lesions or the occurrence of new lesions. Stable disease (SD) was between PR and PD.

### Statistical Analysis

All data were analyzed by SPSS 18.0 software (IBM, Armonk, NY, USA) and GraphPad Prism 5.0 software (GraphPad Software Inc., San Diego, CA, USA). X-tile 3.6.1 software (Yale University, New Haven, CT, USA) was used to determine the optimal cutoff values of continuous variables. The follow-up period is defined as the time from the diagnosis of the disease to the time of the patient's last follow-up or death. Overall survival (OS) is the time from the date of diagnosis to the date of death of any cause. Progression free survival (PFS) is the time from the date of initial treatment to the date of progression or death of any cause. OS and PFS were evaluated with Kaplan–Meier survival analysis. Categorical variables (gender, tumor location, metastatic sites, first-line radiotherapy (yes or no), HCG level (IU/L) (<50/50–5,000/5,000–50,000/50,000 <), LDH level (IU/L) [<1.5 × *N* (upper limit of normal range)/1.5–10 × *N*/10 × *N* <), AFP level (ng/ml) (<10/10–1,000/1,000–10,000/10,000 <) and initial tumor response) and continuous variables (age and tumor markers values) were enrolled in the univariate analysis of OS and PFS for nonseminoma. Log rank test was used for comparisons. Factors that were considered significant in univariate analysis were included in multivariate analysis to identify independent influencing factors. The method of stepwise forward of Cox regression model was used for the multivariate analysis. *P* < 0.05 was considered significant. All *P*-values were two-sided.

## Results

### Patient Characteristics

PMGCT was identified predominantly in young men with 23 male patients and only one female patient. The median age was 29 (16–65) years. Most tumors were located in the anterior mediastinum except for one located in the posterior mediastinum. Pure seminoma was identified in 5 patients and 19 patients were diagnosed as nonseminoma. Large mediastinal masses were visible in images in all patients with the median greatest dimension of tumor being 9.8 (4.1–20) cm.

Chest pain (50%), cough (41.7%), dyspnea (41.7%) were the most common symptoms. Less common symptoms included superior vena cava syndrome (12.5%), weight loss (12.5%), cervical mass (8.3%), and chest wall mass (4.2%).

Lung involvement was found in 4 (16.7%) patients. Nonpulmonary visceral metastases were identified in 3 (12.5%) patients: one patient with liver and bone metastases and two patients with bone metastases. According to the International Germ Cell Cancer Collaborative Group (IGCCCG) classification (14), 19 (79.2%) patients were classified as poor prognosis and 5 (20.8%) were good prognosis (Table 1). β-HCG levels (reference intervals: 0–10.0 IU/L) were elevated in 7 (29.2%) patients with a median β-HCG level of 77.4 (15–15,290) IU/L. LDH levels (reference intervals: 110–240 IU/L) were elevated in 16 (66.7%) patients and the median LDH level was 422 (301–760) IU/L. AFP levels (reference intervals: 0–7.0 ng/mL) were elevated in 17 (70.8%) patients with a median AFP level of 6,097.4 (11.8–444,994) ng/mL.

**Table 1:** Patient characteristics.

**Characteristics**	**Value**
Age (in years) median (range)	29 (16–65)
**Sex**, ***n*** **(%)**
Female	1 (4.2%)
Male	23 (95.8%)
**Histology**, ***n*** **(%)**
Pure seminoma	5 (20.8%)
Teratoma	5 (20.8%)
Yolk sac tumor	7 (29.2%)
Choriocarcinoma	1 (4.2%)
Mixed GCT	6 (25.0%)
**Tumor location**, ***n*** **(%)**
Anterior mediastium	23 (95.8%)
Posterior mediastium	1 (4.2%)
**Metastasis**, ***n*** **(%)**
No	1 (4.2%)
Pleura	15 (62.5%)
Lung	4 (16.6%)
Liver	1 (4.2%)
Bone	3 (12.5%)
**Surgery**, ***n*** **(%)**
Yes	8 (33.3%)
No	16 (66.7%)
**First-line radiotherapy**, ***n*** **(%)**
Yes	12 (50.0%)
No	12 (50.0%)
**IGCCCG classification**, ***n*** **(%)**
Good prognosis	5 (20.8%)
Poor prognosis	19 (79.2%)
**Initial tumor response**, ***n*** **(%)**
CR	8 (33.3%)
PR	4 (16.7%)
SD	7 (29.2%)
PD	5 (20.8%)

*GCT, germ cell tumor; IGCCCG classification, International Germ Cell Cancer Collaborative Group classification (14); CR, complete remission; PR, partial remission; SD, stable disease; PD, progressive disease*.

### Treatment and Outcomes

**Figure 1:**
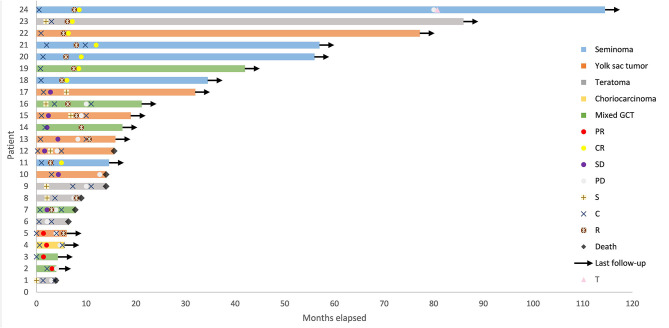
Swimmer Plot depicting time course of primary mediastinal germ cell tumor from diagnosis to death or date of last follow-up. GCT, germ cell tumor; CR, complete remission; PR, partial remission; SD, stable disease; PD, progressive disease; S, surgery; C, chemotherapy; R, radiotherapy; T, autologous hematopoietic progenitor transplantation following high dose chemotherapy.

Nineteen (79.2%) patients received BEP chemotherapy (bleomycin 30 mg on days 1, 8, and 15, etoposide 100 mg/m^2^ on days 1–3, cisplatin 100 mg/m^2^ on days 1–3, every 3 weeks) and 5 (20.8%) patients received surgery as initial treatment of first-line treatment. None of the patients was irradiated at initial treatment. All the five patients who underwent surgery received adjuvant chemotherapy, of which two received BEP, one received VPB (vincristine 1 mg/m^2^ on day 1, cisplatin 70 mg/m^2^ on days 1–2, bleomycin 15 mg on days 1, 8, and 15, every 3 weeks), one received CAO (ifosfamide 3 mg/m^2^ on day 1, epirubicin 80 mg/m^2^ on days 1–2, vincristine 1 mg/m^2^ on day 1, every 3 weeks), and one received TC (paclitaxel 175 mg/m^2^ on day 1, carboplatin AUC = 4 on day 1, every 3 weeks). Eight (33.3%) patients received surgery and 15 (62.5%) patients received radiotherapy in total. Five (20.8%) patients received only chemotherapy. The median total dose of radiotherapy was 46 (25.5–60) Gy. The details are shown in Figure 1.

For patients with seminoma, all five patients showed favorable outcomes with a CR. One patient experienced disease recurrence and received high dose chemotherapy followed by autologous hematopoietic progenitor transplantation. It is noteworthy that there was an increase in LDH concentration at the time of recurrence.

For the optimal tumor response of patients with nonseminoma, three patients achieved CR, four patients achieved PR, seven patients achieved SD, and five patients achieved PD. In total, 13 (68.4%) patients experienced PD, including five patients with yolk sac tumor, three patients with mixed GCT which contained yolk sac elements, four patients with teratoma, and one patient with choriocarcinoma. At the time of PD, elevated AFP was detected in all the five patients with yolk sac tumor, two patients with teratoma and one patient with mixed GCT. Besides, LDH levels increased in one patient with teratoma and HCG levels increased in one patient with choriocarcinoma. Nine patients received salvage chemotherapy alone after disease recurrence. Two patients received salvage chemotherapy in combination with radiotherapy. One patient received radiotherapy alone and one patient did not receive any treatment.

### Survival and Progression

**Figure 2:**
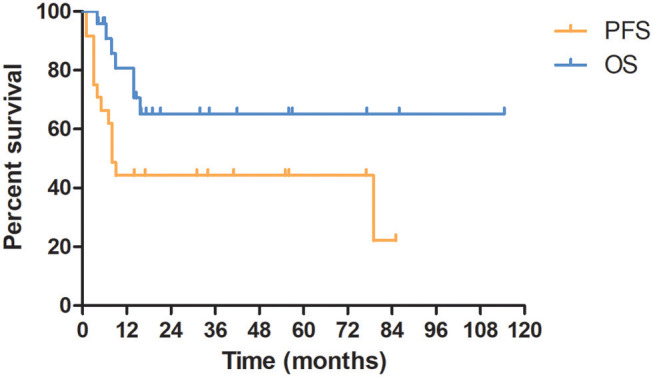
Kaplan–Meier curves of overall survival and progression free survival for all patients.

The median follow-up time was 15.8 (3.9–114.5) months. The 1- and 5-year OS rate for all patients was 80.7 and 65.2%, respectively. Seven (29.2%) patients died. All of the dead patients were diagnosed as nonseminoma and died of progressive disease. The median PFS time was 9.0 months and the 5-year PFS rate was 44.3% (Figure 2). The median survival time after recurrence was 4.3 months and 1-year survival rate after recurrence was 23.4%.

### Prognosis

In the univariate analysis of OS for all patients, a prolonged trend of OS was observed in patients with seminoma (*P* = 0.093). The 5-year OS rate for nonseminoma and seminoma was 54.1 and 100%, respectively. Univariate analysis of PFS showed that PFS of patients with nonseminoma was significantly shorter than that of patients with seminoma (*P* = 0.044). The 5-year PFS rate for nonseminoma and seminoma was 28.7 and 100%, respectively. And the median PFS time for nonseminoma and seminoma were 8.0 and 79.5 months, respectively.

In the univariate analysis of prognostic factors for OS of nonseminoma, a protracted trend of OS was shown in patients with the greatest dimension of tumor <7 cm (*P* = 0.052; Table 2). Patients who received first-line radiotherapy had longer OS time compared with those who did not (*P* = 0.037). In the univariate analysis for PFS of nonseminoma, the greatest dimension of tumor (<7 vs. >7 cm) and first-line radiotherapy influenced PFS significantly (*P* = 0.037 and 0.027, respectively). The greatest dimension of tumor <7 cm and first-line radiotherapy indicated superior PFS. However, neither of them was significant in the multivariate analysis for OS or PFS.

**Table 2:** Univariate analysis of prognostic factors for OS and PFS of patients with nonseminoma.

	**No. of patients**	**2-year OS rate (%)**	***P***	**2-year PFS rate (%)**	***P***
**Greatest dimension of tumor at diagnosis**					
>7cm	13	34.6		18.5	
<7cm	4	100	0.052	75	0.037
Not clear	2				
**First-line radiotherapy**					
Yes	7	85.7		57.1	
No	12	26.2	0.037	10	0.027

*OS, overall survival; PFS, progression free survival*.

## Discussion

Primary EGCTs are rare tumors which most often occur in the midline of the body: mediastinum, retroperitoneum, etc. Their histological attributes are similar to gonadal GCTs, however, their biology, especially nonseminoma, is substantial different (15). Some researchers reported that the prognosis of PMGCTs is the worst of all EGCTs (16, 17). There are few large population studies which enrolled patients with EGCTs and the reports of PMGCTs are fewer still. It has been reported that ~90% of PMGCTs are diagnosed in men aged 20–40, with tumors mostly located in the anterior mediastinum and, very rarely, in the middle or posterior mediastinum (18). In our study, we enrolled 24 Chinese patients with PMGCTs. The tumors were predominantly diagnosed in young men, with one female patient among the 24 total. The tumors were located in the anterior mediastinum for 23 patients, and in the posterior mediastinum for the one patient. The size of tumor is usually large at the moment of diagnosis, especially nonseminoma. And as the tumor grows, it may compress and invade surrounding mediastinal structures. In our analysis, chest pain, cough and dyspnea were the most common symptoms, which were quite atypical and made the disease hard to distinguish.

It has been reported that the prognosis of patients with advanced metastatic testicular seminoma is not different from that of patients with extragonadal seminoma (15). Outcomes of primary cisplatin-based chemotherapy have been favorable and mediastinal seminoma shares the same radiosensitivity as its gonadal counterpart. However, it has also been reported that the prognosis for radiotherapy as initial treatment was worse than primary chemotherapy in seminoma (4). Bokemeyer et al. (2) reported the largest sample of primary mediastinal seminoma so far. They found that liver metastases and the number of metastatic sites had significant influence on survival. The 5-year OS and PFS rates for 51 patients with mediastinal seminoma were 88 and 88%. Besides, IGCCCG (14) reported that the 5-year OS and PFS rates for 41 patients with mediastinal seminoma were 88 and 80%. In our study, all the five patients with seminoma received primary chemotherapy followed by radiotherapy and all but one achieved persistent CR. However, the analysis of prognostic factors was not available due to the small dataset.

Compared with advanced metastatic testicular nonseminoma, the prognosis of patients with primary mediastinal nonseminoma is much worse (15). Furthermore, some studies reported that the prognosis of primary mediastinal nonseminoma is worse than that of primary mediastinal seminoma (3, 4). Some occidental studies reported 40–50% long-term survival for patients with nonseminoma (2, 4, 15, 19, 20). In a multicenter study that enrolled 341 patients with PMGCTs from USA and Europe, Bokemeyer et al. (2, 4) reported the 5-year OS and PFS rates for mediastinal nonseminoma were 45 and 44%, respectively. A more recent study showed the 5-year OS rate was 46% for nonseminomatous patients (21). In the oriental population, Wang et al. (22) reported that the 5-year OS and PFS rates of mediastinal nonseminomatous patients were 49.2 and 32.8%, respectively. Ebi et al. (12) reported 5-year OS and PFS rates for 14 patients with primary mediastinal nonseminoma were 60 and 44%. The 5-year OS and PFS rates for patients with primary mediastinal nonseminoma in our group were 54.1 and 28.7%. While the OS rate of our patients was consistent with previous studies, the PFS rate was substantially lower.

The treatment approach of primary mediastinal nonseminoma is identical to that of metastatic testicular nonseminoma. The use of cisplatin-based chemotherapy has greatly improved the survival rate of patients with primary mediastinal nonseminoma (4). Necrosis has been frequently identified in post-chemotherapy residual tumors, but complete necrosis of tumor is rarely achieved with chemotherapy alone (23) and viable tumor still remains in 30–47% of patients (15). Vuky et al. reported 57% of patients' surgical specimens after chemotherapy showed viable tumor, although the STMs were normal (24). Therefore, post-chemotherapy resection of residual tumor is likely a necessary step to reduce recurrence and improve the efficacy. Nevertheless, most patients with nonseminoma in our study did not receive post-chemotherapy resection because of extensive tumor invasion or poor tolerance, which may be an explanation for the inferior PFS. Besides, previous studies found that yolk sac tumors were less sensitive to chemotherapy and carried a poorer prognosis (25). The high proportion of yolk sac tumors and mixed GCT containing yolk sac elements in our study may also lead to the low PFS rate. It was reported that radiotherapy in a chemotherapy-based treatment regimen was an independent factor that significantly influenced local recurrence and overall survival of patients with primary mediastinal nonseminoma (22). In our analysis, patients with nonseminoma who received first-line radiotherapy had significantly prolonged OS and PFS time compared with those who were not irradiated. Modern cutting-edge techniques of radiotherapy ensure sufficient dose to the tumor volume and limited irradiation to organs at risk. Still, it was reported that some patients experienced ≥ Grade II hematologic toxicity, esophagitis, and pneumonitis. Radiotherapy may also affect the tolerance of patients for the following full-dose chemotherapy (22). We consider that radiotherapy is an alternative treatment for patients with unresectable disease.

It was reported that the treatment outcome of relapsed primary mediastinal nonseminoma is inferior to that of metastatic testicular nonseminoma. The prognosis of patients with primary mediastinal nonseminoma who relapsed was dismal, with a salvage rate of <10% (4, 15, 19, 20). Saxman et al. (26) reported that only 7% of patients with primary mediastinal nonseminoma who received salvage chemotherapy achieved long-term disease-free survival. In our study, most patients with relapsed primary mediastinal nonseminoma were unable to sustain complete remission. The median survival time after recurrence was 4.3 months and the 1-year survival rate was only 23.4% despite salvage treatment. Salvage surgery is controversial. On the one hand, persistent elevation of STM after chemotherapy indicates poor prognosis and it is regarded as a contraindication of surgery. But on the other, the efficacy of salvage chemotherapy is unsatisfactory and few patients achieve long-term disease-free survival (2, 4). Some studies reported that surgery could be a beneficial salvage treatment even when no further chemotherapy is available (3, 24, 27). Radical resection for primary mediastinal nonseminoma is challenging in most cases. Dense adhesion and invasion of surrounding structure, especially great vessels, make radical surgery impossible in many cases. Furthermore, the morbidity and mortality of the complications of surgery were noticeable due to extensive thoracic resection (8). Albany et al. recommended four cycles of etoposide, ifosfamide, and cisplatin (VIP) instead of BEP to prevent pulmonary complications. If surgery is not feasible, high-dose chemotherapy followed by stem cell transplant may be an effective approach (15, 19, 20).

It has been reported that 6% of patients with primary mediastinal nonseminoma develop hematological malignancies. Acute megakaryoblastic leukemia and myelodysplasia are the most common ones. The prognosis of the hematological malignancies is dismal with a median survival time of only 5–6 months (15, 28). Additionally, some researchers indicated that metachronous testicular cancers develop in 10.3% of patients ten years after the diagnosis of EGCT. The risk of patients with retroperitoneal or nonseminomatous tumors was relatively higher (4, 29). During the follow-up period of our patients, no second tumor was identified.

In conclusion, this study is one of the few studies of Asian patients with PMGCT which investigated the characteristics of the rare disease and treatment outcomes. It should be noted, however, that this is a single-center retrospective study with a limited sample size. Although the analysis of influencing factors of the prognosis was based on a small sample size, it may provide a reference for future research and clinical practice. Further multicenter, randomized, and controlled investigations are needed to guide how to sequence and combine treatment modalities to improve the prognosis of patients especially those with primary mediastinal nonseminoma.

## Data Availability Statement

The raw data supporting the conclusions of this article will be made available by the authors, without undue reservation.

## Ethics Statement

The studies involving human participants were reviewed and approved by Institutional Ethics Committee of Peking University Cancer Hospital. Written informed consent to participate in this study was provided by the participants' legal guardian/next of kin.

## Author Contributions

LW and JZho were responsible for the collection of clinical data. LW drafted the manuscript. JZho directed the writing. JZha, TA, YW, MZ, MW, ZW, JL, XY, HC, and JZho were responsible for the supplement and refinement of clinical data and collectively carried out treatment procedures. All authors read and approved the final manuscript.

## Conflict of Interest

The authors declare that the research was conducted in the absence of any commercial or financial relationships that could be construed as a potential conflict of interest.

## Abbreviations

PMGCTs: primary mediastinal germ cell tumors
OS: overall survival
PFS: progression free survival
CR: complete response
PR: partial response
GCTs: germ cell tumors
EGCTs: extragonadal germ cell tumors
STMs: serum tumor markers
LDH: lactate dehydrogenase
AFP: alpha-fetoprotein
β-HCG: beta-human chorionic gonadotropin
CT: computed tomography
PD: progressive disease
SD: stable disease
IGCCCG: International Germ Cell Cancer Collaborative Group
BEP, bleomycin, etoposide: and cisplatin.
